# Voretigene Neparvovec Gene Therapy in Clinical Practice: A 12-Month, Single-Center, In-Depth Analysis of Beneficial and Adverse Drug Effects

**DOI:** 10.1167/tvst.15.5.9

**Published:** 2026-05-12

**Authors:** Francesco Testa, Valentina Di Iorio, Paolo Melillo, Marianthi Karali, Clemente Maria Iodice, Pisani Danila, Claudio Iovino, Sandro Banfi, Settimio Rossi, Michele Della Corte, Francesca Simonelli

**Affiliations:** 1 Eye Clinic, Multidisciplinary Department of Medical, Surgical and Dental Sciences, University of Campania ‘Luigi Vanvitelli’, Naples, Italy; 2 Medical Genetics, Department of Precision Medicine, University of Campania ‘Luigi Vanvitelli’, Naples, Italy; 3 Telethon Institute of Genetics and Medicine, Pozzuoli, Italy

**Keywords:** voretigene neparvovec, *RPE65* gene, inherited retinal dystrophies, chorioretinal atrophy, gene therapy

## Abstract

**Purpose:**

To investigate the effects of voretigene neparvovec (VN) treatment in patients with *RPE65*-related retinal dystrophies, with particular focus on chorioretinal atrophy (CRA) and its impact on visual function.

**Methods:**

Twenty-five patients treated with VN were evaluated over a 12-month follow-up and stratified according to the presence of postoperative CRA, classified into nummular, mixed and touchdown patterns.

**Results:**

Best-corrected visual acuity (BCVA) improved by a median of 11 Early Treatment Diabetic Retinopathy Study (ETDRS) letters (*P* < 0.001). Semiautomated kinetic visual field (SKVF) showed a significant (*P* < 0.001) enlargement for both I4e and III4e stimulus sizes. Fullfield stimulus threshold (FST) results also improved significantly (*P* < 0.001), with median changes of −2.79 (−3.61 to −2.11) log cd·s/m^2^ for white, −3.02 (−4.05 to −2.24) log cd·s/m^2^ for blue, and −1.30 (−1.76 to −0.74) log cd·s/m^2^ for red stimuli. Fifteen patients developed CRA (60%), of whom nine exhibited progressive nummular/mixed CRA, which significantly expanded over time (*P* < 0.001), and six showed the localized, non-progressive touchdown pattern. Younger age and a clinical diagnosis of LCA were significantly associated with nummular/mixed CRA. No significant differences in BCVA or FST improvements were found between nummular/mixed and touchdown/no-CRA groups. Conversely, significant SKVF I4e enlargement occurred only in the touchdown/no-CRA subgroup (1624°^2^, 0−6278; *P* < 0.001), with a significant intergroup difference (*P* = 0.003).

**Conclusions:**

Our findings confirmed the visual function improvements following VN and the relatively high prevalence of CRA, which seems to impact negatively only on SKVF enlargement.

**Translational Relevance:**

These findings are useful to better understand risks and benefits of VN gene therapy.

## Introduction

Inherited retinal dystrophies (IRDs) are a heterogeneous group of genetic disorders characterized by progressive retinal degeneration causing visual loss in affected individuals. The great variability and complexity of IRDs clinical presentation are due to the genetic heterogeneity matched with the phenotypic diversity.^1^ To date, over 280 genes have been identified in various forms of IRDs, and biallelic mutations in the *RPE65* gene have been associated with sight-threatening autosomal recessive disorders including early-onset severe retinal dystrophy (EOSRD) and Leber congenital amaurosis (LCA).^2^

Specifically, *RPE65* gene mutations account for approximately 5% of cases of severe IRDs and cause an early-onset and severe rod-mediated retinal disease characterized by a progressive and profound loss of vision.^3^ Phase I, II, and III clinical trials have demonstrated the safety and efficacy of a recombinant adeno-associated viral vector serotype 2 (AAV2) containing a therapeutic *RPE65* gene sequence delivered by subretinal injection.^4^^,^^5^ In 2017, the U.S. Food and Drug Administration and subsequently the European Medicines Agency approved voretigene neparvovec (VN) as the first gene augmentation therapy in humans for biallelic *RPE65-*mediated IRDs.

Despite its clinical success in improving vision, one concern that has emerged from post-marketing surveillance is the degeneration or loss of both the retina and the underlying choroid, known as chorioretinal atrophy (CRA).^6^ In natural history studies,^7^^–^^10^ atrophic changes, including lacunar CRA in the periphery, have been reported in relatively few cases with advanced untreated disease, whereas recent clinical studies^11^^–^^15^ have reported a high incidence of CRA in patients treated with VN ranging between 14.4% and 50%. These studies have sought to provide insights into the mechanisms of CRA following treatment with VN, investigating several key factors that may contribute to retinal degeneration. These include surgical trauma, immune responses to the AAV2 vector, vector-related toxicity, and overexpression of the therapeutic gene, which could induce cellular stress and further exacerbate retinal damage.^11^^–^^15^ Although most of the previous studies have shown that CRA does not significantly affect visual function improvement,^12^^,^^13^^,^^15^^–^^17^ this phenomenon raises concerns about the long-term safety and efficacy of gene therapy. In this study, we aimed to analyze functional, morphological, and surgical features of patients treated with VN, focusing on CRA onset and its impact on visual function over 1-year post-treatment follow-up.

## Methods

### Study Design and Participants

This retrospective chart review study included patients with biallelic *RPE65*-IRD treated with VN at the Centre for Rare Ocular Diseases of the University of Campania Luigi Vanvitelli, Naples, Italy. All patients gave their consent to participate in the post-approval study by Novartis, the VN licensor in the European Union (EU). All procedures adhered to the tenets of the Declaration of Helsinki and local regulations. Ethics approval was obtained from the Local Ethics Committee.

After a discussion regarding the pros and cons of VN treatment, all participants or their legal guardians or parents provided written informed consent. All patients included in the study had a minimum post-treatment follow-up of 1 year and satisfied the criteria for treatment with VN: (1) clinical diagnosis of LCA or EOSRD; (2) *RPE65* biallelic mutations (biallelism was confirmed by segregation analysis); (3) adults or children 3 years or older; (4) best-corrected visual acuity (BCVA) equal to or worse than 0.5 logarithm of the minimum angle of resolution (logMAR); and (5) central retinal thickness thicker than 100 µm.

LCA diagnosis was based on severe visual impairment from birth (or from the first months of life), roving eye movements or nystagmus, poor pupillary light responses, oculodigital sign, and undetectable or severely abnormal fullfield electroretinography (ERG).^2^ On the other hand, EOSRD featured later onset visual impairment (typically after infancy, but before 5 years of age), with variably preserved visual acuity and minimally preserved fullfield ERG.^2^

### Clinical and Imaging Data

A complete ophthalmological evaluation was performed at baseline and at each scheduled post-treatment follow-up visit—namely at day 30 and day 45 (for the second and first treated eye, respectively), month 6, and month 12. With the exception of the earliest postoperative visit (day 30/day 45), for which data were available in 35 out of 41 eyes (85%), all other assessments were completed in all 41 treated eyes. In the current study, the effects of VN treatment on visual function were assessed using BCVA, fullfield stimulus threshold (FST) test, semiautomated kinetic visual field (SKVF). BCVA was measured using standard protocol Early Treatment Diabetic Retinopathy Study (ETDRS) charts; letter scores were converted to logMAR.

SKVF testing was performed using an Octopus 900 Pro perimeter with Eye Suite i4.000 software (Haag-Streit, Koeniz, Switzerland). Background illumination of the bowl was 31.4 apostilb. Data were collected using stimulus test sizes V4e, III4e, and I4e; the total seeing area was calculated for each isopter (seeing area minus defined scotoma) and expressed as square degree (°^2^). Test vectors were presented approximately every 15°, at an angular velocity of 4°/s and originating approximately 10° outside the age-correlated normal isopter. Scotomas were mapped using an angular velocity of 2°/s, originating from the assumed center using at least 12 vectors. Blind spot mapping was done with the smallest test size target that the patient was able to see.

FST testing, performed with the Espion E3 ColorDome (Diagnosys, Cambridge, UK), measured luminance thresholds using fullfield stimuli generated by narrowband light-emitting diodes (LEDs). Stimuli below 0.01 cd·s/m^2^ (“dim” LEDs) had peak wavelengths of 468 nm (blue) and 632 nm (red); stimuli above 0.01 cd·s/m^2^ (“bright” LEDs) were 444 nm (blue) and 632 nm (red). A simple detection paradigm with one-button box was used: Subjects pressed a response button to indicate whether a brief fullfield flash was perceived, without an alternative forced-choice procedure. Pupils were dilated before testing, and FST testing was performed in the dark after 40 minutes of dark adaptation, beginning with blue stimuli, followed by 6500K white and red stimuli. FSTs were analyzed in absolute luminance units (log cd·s/m^2^). An isolated rod-mediated response was defined as a blue–red threshold difference ≥ 2.0 log units in favor of the blue stimulus, consistent with the expected spectral separation under scotopic conditions.

Furthermore, changes in retinal morphology were evaluated by indirect ophthalmoscopy, slit-lamp examination, color fundus imaging, and spectral-domain optical coherence tomography (SD-OCT). Fundus imaging was acquired with a true color fundus scanner camera (iCare EIDON; CenterVue, Padua, Italy) or an ultra-widefield pseudocolor imaging system (Optos, Dunfermline, UK). SD-OCT scans were acquired with the SPECTRALIS OCT (Heidelberg Engineering, Heidelberg, Germany) with a dense 20° × 15° volume scan with the follow-up function at each time point or with a linear scan in the case of unfeasibility of dense volume scan (e.g., because of nystagmus). Moreover, the presence or the absence of a detectable ellipsoid zone (EZ) band was evaluated within the foveal region on B-scans of the volume scan (available in 15 patients) or on the linear scan centered in fovea (in 10 patients with nystagmus for whom dense volume scans were not possible). Finally, intraocular pressure was measured with Goldmann applanation.

Considering the recent description of CRA in real-life studies,^6^^,^^11^^,^^15^^,^^18^ all retinal fundus images were reviewed in order to assess postoperative CRA, defined as newly emerged areas of retinal atrophy with evident loss of retinal pigment epithelium (RPE) (i.e., a variable degree of translucency with a detectable choroidal vasculature). Moreover, CRA was further classified in three patterns according to the imaging characteristics: nummular CRA, predominantly affecting the periphery; mixed pattern, with confluent nummular areas and perifoveal CRA; and touchdown CRA, corresponding to the atrophy at the injection site.^15^

The atrophic lesions were manually segmented by two independent expert graders using ImageJ 2.1.0/1.53c (National Institutes of Health, Bethesda, MD).^19^ The two graders were masked to all clinical outcomes. After independent segmentation, the two sets of lesion maps were compared, and complete agreement was reached for both lesion count and lesion boundaries in all cases. For this reason, separate quantitative measurements from each grader and formal interobserver agreement statistics were not generated. Finally, lesion areas were converted to millimeters by scaling to an optic disc diameter of 1.8 mm, based on the Age-Related Eye Disease Study 2 (AREDS2) definition, as previously reported,^15^^,^^20^ and the total CRA area was then calculated for each eye.

### Surgical Aspects

A single experienced vitreoretinal surgeon (MDC) performed all surgeries according to the general guidelines of the recommended protocol reported by Russell et al.^5^ Specifically, a 25-gauge vitrectomy (CONSTELLATION Vision System; Alcon, Geneva, Switzerland) was performed under general anesthesia in all patients except two siblings. In these siblings with glucose-6-phosphate dehydrogenase deficiency, retrobulbar anesthesia was preferred.

After a standard core vitrectomy, posterior vitreous detachment was induced with preservative-free triamcinolone acetonide staining. A total volume of 300 µL was manually delivered into the subretinal space by a single bleb or multiple blebs of VN along the upper vascular arcades, avoiding the retinal vessels and areas of atrophy, using a 25/38-gauge needle (Cannula PolyTip 25g/38g; MedOne Surgical, Sarasota, FL). The subretinal injection procedure was conducted under the guidance of intraoperative optical coherence tomography (OCT) using either a Proveo 8 Ophthalmic Microscope (Leica Microsystems, Wetzlar, Germany) or an OPMI LUMERA 700 with RESCAN 700 (Carl Zeiss, Oberkochen, Germany)*.* Although the injection strategy was the same in all eyes, the bleb direction was unpredictable. The evaluation of intraoperative foveal detachment and the number of retinal blebs was performed retrospectively reviewing both the microscope video and the intraoperative OCT recordings.

A peripheral retinal check with scleral indentation was performed to identify any pre-existent or iatrogenic retinal defect including holes/tears, followed by an air/fluid exchange. Finally, sclerotomies were sutured with Vicryl 7/0, and subconjunctival injection of betamethasone and gentamicin was administered*.* Patients received 1 mg/kg/d (up to 40 mg/d) of oral prednisone for 7 days, starting 3 days before the surgery. Prednisone was tapered (0.5 mg/kg/d, up to 20 mg/d) for the subsequent 7 days or until 3 days before the injection in the second eye. All intra- or postoperative complications were recorded.

### Statistical Analysis

The statistical analysis was performed using SPSS Statistics 21.0.0.0 (IBM, Chicago, IL). Both treated eyes of single patients were included in the analysis when data were evaluable. Descriptive statistics were calculated and reported as median (interquartile range [IQR]) for continuous variables and count (frequency) for categorical variables. Generalized estimating equations (GEEs) were applied using an appropriate covariance structure, as previously described by Glynn and Rosner.^21^ This semi-parametric approach was selected because it accounts for both inter-eye correlation and within-eye longitudinal correlation, it does not require normally distributed data, and it ensures robustness despite occasional missing measurements.

Comparisons between follow-up visits and baseline were performed at the first post-treatment time point (i.e., approximately 45 days, or 30 days after treatment of the first and second eye, respectively) and at approximately 6 and 12 months after treatment. To explore factors associated with CRA development and its potential impact on treatment response, patients with progressive CRA patterns (nummular or mixed) were compared with those showing either no CRA or the localized and stable touchdown pattern. No formal correction for multiple testing was applied, as this was an exploratory real-world study aimed at characterizing functional and structural trends after VN; therefore, results were interpreted with caution and emphasis was placed on the consistency of findings across outcomes rather than on isolated *P* values.

## Results

The study included 25 patients (41 eyes) with a median age of 19 years (IQR = 10–43 years; range, 6–66 years). Table 1 summarizes the main demographic, clinical, and genetic characteristics of the cohort. The sample included 11 males (44%) and 14 females (56%). ERG responses were non-detectable in all patients, and nystagmus was present in the majority of patients (17/25, 68%). Sixteen individuals were treated in both eyes, with a maximum interval of 2 months between the two surgical procedures (median, 14 days). Six patients underwent unilateral VN treatment because the fellow eye did not meet eligibility criteria for VN treatment due to advanced atrophy and limited residual structure (*n* = 4) or BCVA better than 0.5 logMAR (*n* = 2). The other three patients underwent bilateral treatment, but the second eye was treated approximately 1 year after first eye and 1-year follow-up data were not yet available at the time of the chart review of this study and therefore not included.

**Table 1.** Main Demographics, Clinical, and Genetic Features of the Treated *RPE65* Patients

ID	Age (Y)	Gender	Clinical Diagnosis	Variants in *RPE65* Gene	
1	9	M	EOSRD	c.938A>G	c.1445A>T
2	8	F	LCA	c.938A>G	c.1102T>C
3^*^	8	F	LCA	c.1229C>A	c.1102T>C
4^*^	6	F	LCA	c.1229C>A	c.1102T>C
5	11	M	EOSRD	c.370C>T	c.1543C>T
6	9	F	EOSRD	c.1543C>T	c.1555G>T
7	18	F	LCA	c.1210_1211insCTGG	c.1206G>A
8	11	M	LCA	c.11+5G>A	c.700C>T
9	10	M	LCA	c.94G>T	c.94G>T
10	34	F	EOSRD	c.138del	c.138del
11	16	F	EOSRD	c.65T>C	c.10C>T
12	20	F	LCA	c.762G>T	c.762G>T
13^†^	30	M	LCA	c.1444G>T	c.1444G>T
14^†^	20	M	LCA	c.1444G>T	c.1444G>T
15	62	F	EOSRD	c.1102T>C	c.1102T>C
16	48	F	EOSRD	c.495+5G>C	c.495+5G>C
17	52	M	LCA	c.11+5G>A	c.1112C>T
18	66	F	EOSRD	c.446C>A	c.446C>A
19	43	F	LCA	c.65T>C	c.893del
20	43	M	LCA	c.271C>T	c.271C>T
21	58	F	LCA	c.370C>T	c.11+5G>A
22	9	M	LCA	c.201_202delinsTT	c.201_202delinsTT
23	10	M	EOSRD	c.1067dup	c.1547C>T
24	37	F	EOSRD	c.493C>T	c.493C>T
25	31	M	LCA	c.138del	c.138del

* Two sisters.

† Two brothers.

As summarized in Table 2, all functional parameters showed significant improvement after treatment across the follow-up period. BCVA improved significantly at the first post-treatment visit (*P* < 0.001), with a median gain of 0.12 logMAR (corresponding to approximately 6 ETDRS letters). An additional improvement of 5 letters was observed at the subsequent time points, remaining stable through month 12. SKVF also demonstrated an early and significant expansion (*P* < 0.01) at day 30/day 45 for both I4e and III4e targets, with the enlargement maintained at 12 months. White-light FSTs improved significantly at the first post-treatment assessment (*P* < 0.001) and continued to improve at the 6- and 12-month time points (*P* < 0.001). Similar significant gains (*P* < 0.001) were observed for blue and red stimuli, although the magnitude of improvement for red-light sensitivity was smaller compared to white and blue stimuli. Furthermore, two eyes (4.9%) exhibited an isolated rod-mediated response at baseline, and this condition persisted at the last follow-up, whereas 24 eyes (58.5%) converted from a mixed or cone-mediated response at baseline to an isolated rod-mediated response at the last follow-up. The remaining 15 eyes (36.6%) retained a mixed or cone-mediated response throughout the study period.

**Table 2.** Change of the Functional Parameters Over the 12-Month Follow-Up

Parameter	Time Point	Value, Mean (IQR)	Change From Baseline, Mean (IQR)	*P* ^*^
BCVA (logMAR)	Baseline	1 (0.7 to 1.83)		
	Day 30/day 45	0.7 (0.55 to 1.23)	−0.12 (−0.2 to −0.07)	**<0.001**
	Month 6	0.72 (0.55 to 1.45)	−0.2 (−0.3 to −0.11)	**<0.001**
	Month 12	0.7 (0.5 to 1.21)	−0.2 (−0.29 to −0.02)	**<0.001**
FST, white (log cd·s/m^2^)^†^	Baseline	−1.87 (−2.29 to −1.44)		
	Day 30/day 45	−4.33 (−4.7 to −3.46)	−2.37 (−3.14 to −1.48)	**<0.001**
	Month 6	−4.11 (−5.01 to −2.88)	−2.15 (−2.9 to −1.17)	**<0.001**
	Month 12	−4.86 (−5.22 to −3.87)	−2.79 (−3.61 to −2.11)	**<0.001**
FST, blue (log cd·s/m^2^)^†^	Baseline	−2.13 (−2.67 to −1.69)		
	Day 30/day 45	−5.05 (−5.44 to −3.85)	−2.81 (−3.33 to −1.75)	**<0.001**
	Month 6	−4.83 (−5.61 to −3.52)	−2.61 (−3.48 to −2.02)	**<0.001**
	Month 12	−5.51 (−6.05 to −4.37)	−3.02 (−4.05 to −2.24)	**<0.001**
FST, red (log cd·s/m^2^)^†^	Baseline	−1.59 (−2.36 to −1.01)		
	Day 30/day 45	−2.8 (−3.18 to −2.11)	−1.22 (−1.63 to −0.7)	**0.003**
	Month 6	−2.81 (−3.39 to −2.27)	−1.09 (−1.94 to −0.49)	**<0.001**
	Month 12	−3.11 (−3.37 to −2.33)	−1.3 (−1.76 to −0.74)	**<0.001**
SKVF, I4e (°^2^)	Baseline	0 (0 to 474)		
	Day 30/day 45	506 (0 to 2555)	0 (0 to 1938)	**0.005**
	Month 6	0 (0 to 2260)	0 (0 to 1534)	**0.003**
	Month 12	0 (0 to 2156)	0 (0 to 2062)	**0.006**
SKVF, III4e (°^2^)	Baseline	2274 (0 to 7581)		
	Day 30/day 45	7987 (1130 to 11971)	2529 (0 to 3505)	**<0.001**
	Month 6	4624 (2030 to 11095)	1217 (0 to 3728)	**<0.001**
	Month 12	6599 (2633 to 11027)	1841 (0 to 4469)	**0.004**
SKVF, V4e (°^2^)	Baseline	9725 (1173 to 13130)		
	Day 30/day 45	13031 (5869 to 15140)	1344 (208 to 2514)	0.346
	Month 6	11375 (4802 to 15231)	1486 (−407 to 3608)	**0.024**
	Month 12	12322 (4899 to 14796)	1769 (0 to 3622)	0.706

Day 30/day 45 data were available in 35 of 41 eyes (85.4%).

* *P* values were obtained by generalized estimating equation regression models; statistically significant *P* values are shown in bold.

† More negative FST values indicate improved retinal sensitivity.

With regard to relevant ocular adverse events, one patient (ID 22) presented with inflammatory cyclitic membrane in the right eye the day after treatment; it resolved spontaneously approximately 1 week later, with no therapy other than the standard perioperative corticosteroid regimen administered to all patients. Another patient (ID 2) had a subretinal hemorrhage in the injection site during the treatment of the left eye which spontaneously resolved. One patient (ID 9) experienced a peripheral retinal break during VN delivery which was promptly treated with intraoperative retinal laser. All patients complied with the tapered corticosteroid treatment that was well tolerated, and no transient or sustained elevations in intraocular pressure were detected in any treated eye at any follow-up visit.

**Figure 1.**
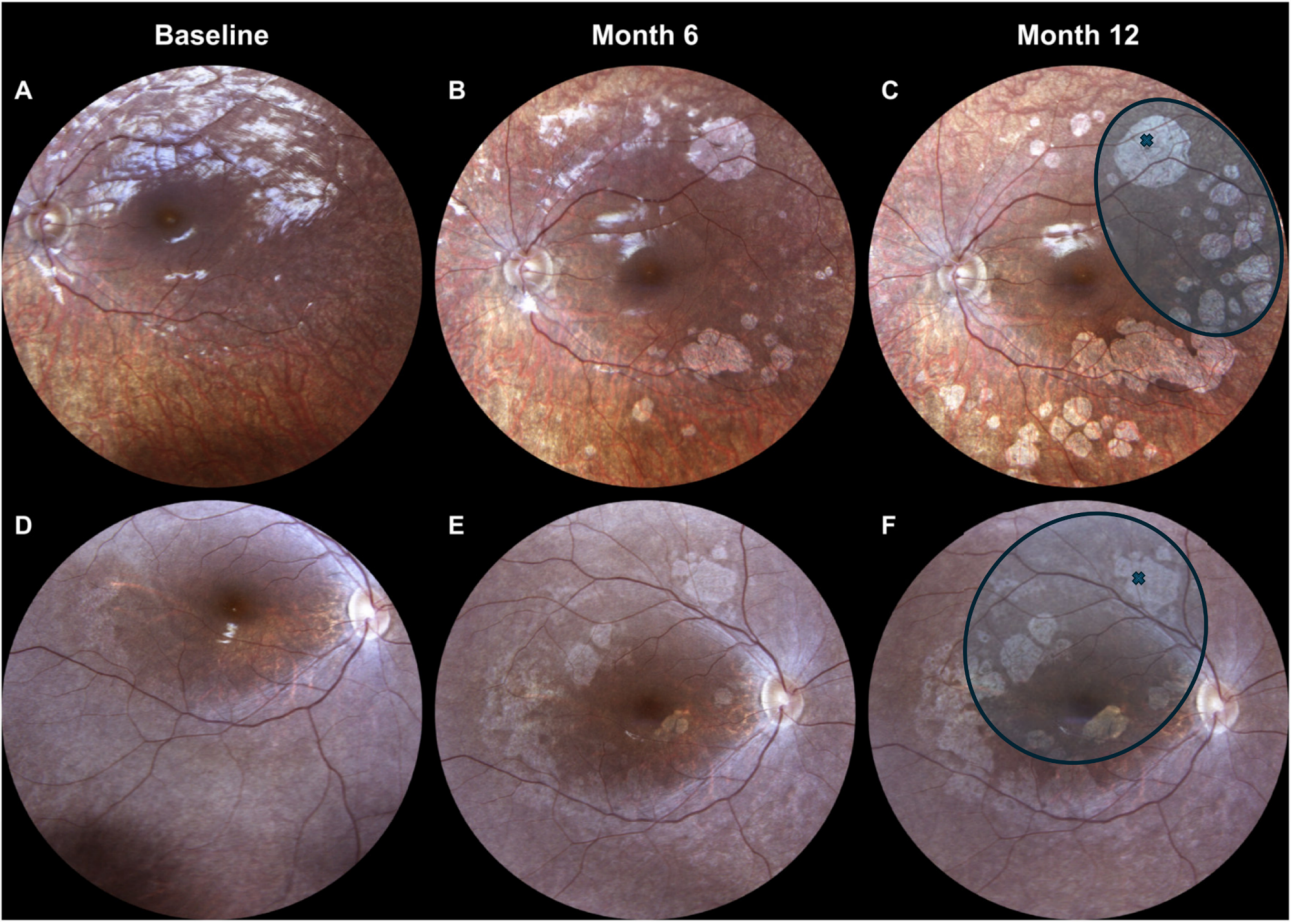
Color fundus photographs of two patients developing progressive CRA after subretinal delivery of VN. (**A**–**C**) In the first patient, a nummular pattern of CRA emerged in the posterior pole and extended toward the mid-periphery over time. (**D**–**F**) In the second patient, a mixed CRA pattern developed, characterized by confluent nummular lesions in the posterior pole and mid-periphery together with additional perifoveal atrophic changes. In panels (**C**) and (**F**), the retinotomy site (*cross*) and the subretinal bleb area (*circled*) are annotated, highlighting that CRA lesions developed both within and outside the bleb.

**Figure 2.**
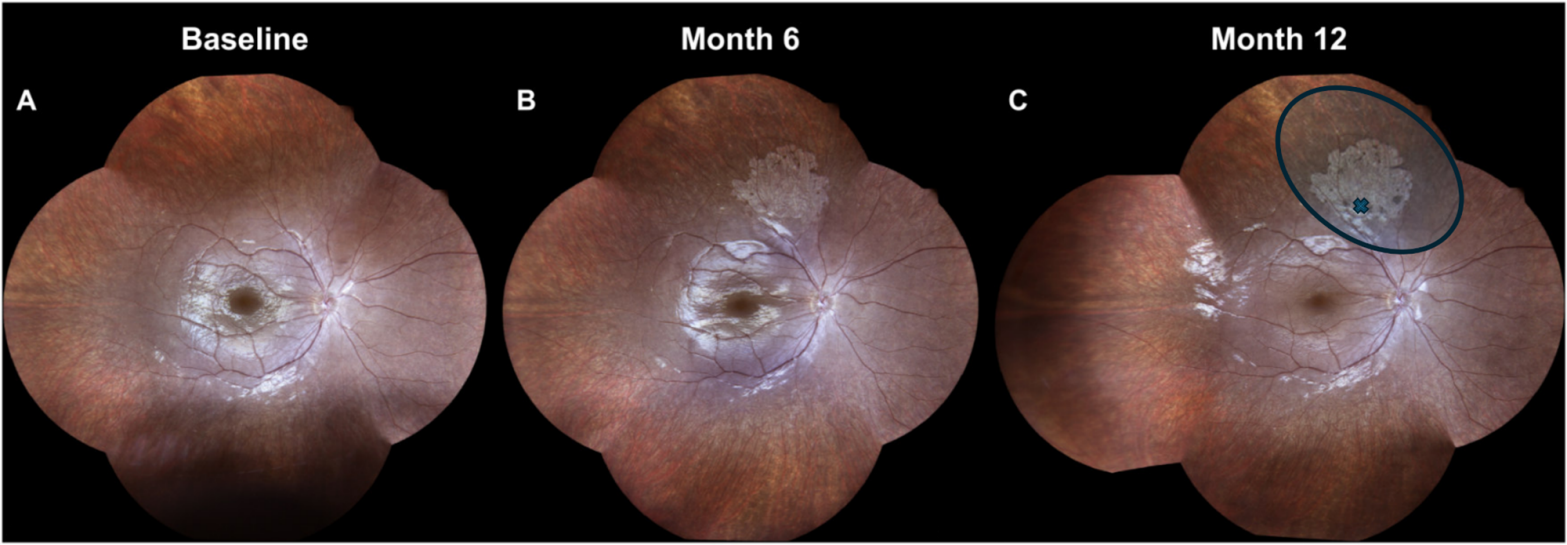
Widefield color fundus photographs of a patient developing a touchdown CRA at the retinotomy site following subretinal VN injection. (**A**–**C**) Compared to baseline (**A**), a focal well-demarcated atrophic lesion emerged at month 6 (**B**) and persisted at month 12 (**C**). In (**C**), the retinotomy site (*cross*) and the subretinal bleb extension (*circled*) are shown, indicating that the touchdown CRA lesion developed within the bleb area.

A total of 15 patients showed CRA (60%). In detail, six patients (ID 1, 2, 5, 6, 12, 18) displayed an area of focal atrophy at the injection site (namely, touchdown CRA), eight patients (ID 3, 4, 7, 8, 11, 13, 14, 25, including two pairs of siblings) developed a nummular area of CRA, predominantly involving the periphery, in both eyes, and one patient (ID 22) showed a mixed pattern (i.e., confluent pattern of nummular and perifoveal atrophy) approximately 6 months after treatment in both eyes. Subsequently, we observed a statistically significant (*P* < 0.001) growth of the atrophic area in the nummular and mixed pattern from 9.9 mm^2^ (IQR = 4.5–15.2 mm^2^) measured at the 6-month time point to 19.2 mm^2^ (IQR = 14.5–28.4 mm^2^) at the 12-month time point. In contrast, the area of touchdown CRA remained quite stable over the 1-year follow-up (6-month time point: 1.6 mm^2^, IQR = 0.7–8.1 mm^2^; 12-month time point: 1.2 mm^2^, IQR = 0.7–8.1 mm^2^; *P* > 0.05). Furthermore, as summarized in Table 3, we analyzed the relationship between CRA and bleb area and found that almost all nummular and mixed CRA fell within and outside the blebs. Figure 1 illustrates the onset and progression of CRA in representative cases with nummular and mixed CRA patterns that fell within and outside the blebs. Figure 2 shows an example of a touchdown CRA, where the focal area of atrophy corresponds to the retinotomy site and fell within the bleb area. Given the stability of touchdown CRA over the 1-year follow-up, we explored potential predictors of progressive CRA by comparing baseline demographic and clinical features between eyes developing nummular/mixed CRA and those showing touchdown or no CRA. Table 4 reports the GEE logistic models fitted to quantify these associations, particularly to estimate odds ratios (ORs) accounting also for inter-eye correlation. Development of progressive nummular/mixed CRA patterns was associated with younger age (*P* = 0.010) and clinical diagnosis of LCA (*P* = 0.047), whereas no significant association was found with the selected functional parameters before treatment. Furthermore, we observed that eyes developing nummular/mixed CRA showed detectable EZ bands more frequently (16/18, 88.8%) compared to those with touchdown/no CRA (13/23, 56.5%), but this association was not statistically significant (*P* = 0.228).

**Table 3.** Relationship Between CRA Patterns and the Subretinal Bleb Area

	Eyes With Touchdown CRA (*n* = 11)	Eyes With Nummular CRA (*n* = 16)	Eyes With Mixed CRA (*n* = 2)
Within bleb area, *n*	9	0	0
Within and outside bleb area, *n*	0	10	2
Outside bleb area, *n*	0	2	0
Not evaluated,^*^ *n*	2	4	0

* Video of surgical procedure was not recorded.

**Table 4.** GEE Logistic Models Evaluating Baseline Predictors of Progressive (Nummular/Mixed) CRA

Feature	Eyes With Nummular or Mixed CRA (*n* = 18)^*^	Eyes With Touchdown or No CRA (*n* = 23)^*^	OR (95% CI)^†^	*P* ^†^
Age (y)	15.6 (9.1 to 22.1)	20.4 (9.1 to 43)	0.944 (0.903 to 0.986)	**0.010**
Clinical diagnosis of LCA	16 (88.8%)	10 (43.5%)	10.286 (1.03 to 102.753)	**0.047**
Male	10 (55.5%)	9 (39.1%)	2.083 (0.396 to 10.948)	0.386
BCVA (logMAR)	1 (0.7 to 1.4)	1 (0.7 to 2.4)	0.991 (0.977 to 1.005)	0.206
SKVF, I4e area (°^2^)	0 (0 to 0)	0 (0 to 2751.8)	—^‡^	—^‡^
SKVF, III4e area (°^2^)	800 (0 to 6280)	3811 (0 to 7727)	1 (1 to 1)	0.524
SKVF, V4e area (°^2^)	5423 (592 to 12789)	10544 (1556 to 14429)	1 (1 to 1)	0.695
FST, white (log cd·s/m^2^)^§^	−1.91 (−2.34 to −1.35)	−1.83 (−2.26 to −1.54)	1 (0.999 to 1.001)	0.975
FST, blue (log cd·s/m^2^)^§^	−2.35 (−3.04 to −1.81)	−1.98 (−2.5 to −1.48)	1 (0.999 to 1.001)	0.732
FST, red (log cd·s/m^2^)^§^	−1.44 (−1.84 to −1.01)	−1.83 (−2.47 to −0.98)	1.003 (0.996 to 1.009)	0.394
Present EZ band	16 (88.8%)	13 (56.5%)	1.003 (0.998 to 1.007)	0.228
Absent EZ band	2 (11.1%)	10 (43.5%)	0.997 (0.993 to 1.002)	

* Data are reported as median (IQR) or count (frequency) for continuous and categorical variables, respectively.

† ORs, relative 95% CIs, and *P* values were obtained by GEE logistic regression models; statistically significant *P* values are shown in bold.

^‡^Not estimable by the specified GEE model.

§ More negative FST values indicate improved retinal sensitivity.

The analysis of surgical parameters showed no significant association between the development of nummular/mixed CRA and the number of blebs (OR = 1.00; 95% confidence interval [CI], 0.999–1.00; *P* = 0.443), the injected volume (OR = 1.00; 95% CI, 1.00–1.00; *P* = 0.820), or the duration of surgery (OR = 1.00; 95% CI, 1.00–1.00; *P* = 0.338). Furthermore, eyes experiencing intraoperative foveal detachment were more likely to develop nummular/mixed CRA (10/18, 58.8%) than those without intraoperative foveal detachment (4/23, 17.3%). However, after accounting for inter-eye correlation using the GEE model, this association did not reach statistical significance (OR = 1.00; 95% CI, 0.999–1.00; *P* = 0.059).

**Figure 3.**
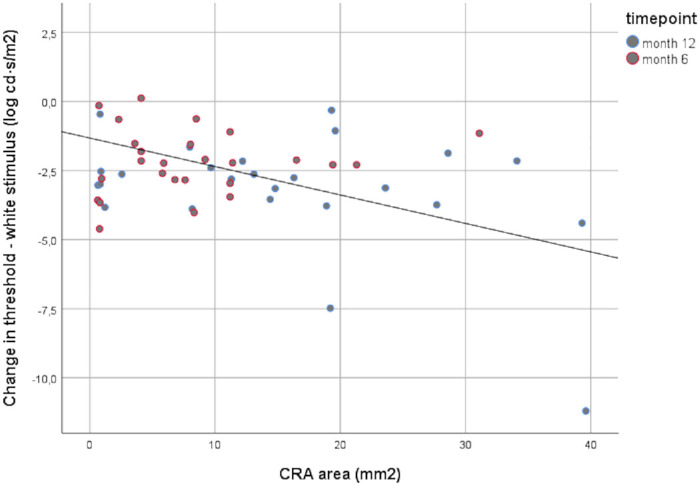
Relationship between changes in FSTs to white-light stimuli and nummular or mixed CRA area evaluated in eight patients.

Finally, as summarized in Table 5, we compared the changes at the 12-month follow-up time point between the two subgroups. In both subgroups, BCVA and FSTs showed significant improvements (*P* < 0.05) with no significant inter-group differences (*P* > 0.20). In contrast, SKVF showed significant improvement in the touchdown/no CRA group (*P* < 0.05) but not in the nummular/mixed CRA group, with a statistically significant (*P* = 0.03) inter-group difference for the I4e area. Analyzing the progression of the area of nummular and mixed CRA (evaluated in eight patients), we did not observe any significant relationship with changes in the selected functional parameters, except for FST white-light sensitivity thresholds. Actually, as shown in Figure 3, changes in FST white-light sensitivity thresholds were significantly inversely correlated (β = −0.103; *P* < 0.001) with the measured CRA area.

**Table 5.** Comparison of Eyes With and Without CRA at the End of the 12-Month Follow-Up

	Eyes With Nummular or Mixed CRA (*n* = 18)^*^		Eye With Touchdown or No CRA (*n* = 23)^*^		
Change at 12 Months Compared to Baseline	Median (IQR)	*P* ^*^	Median (IQR)	*P* ^*^	*P* (Intergroup)
BCVA (logMAR)	−0.19 (−0.28 to −0.02)	<0.001	−0.20 (−0.30 to −0.02)	<0.001	0.201
SKVF, I4e area (°^2^)	0 (0 to 100)	0.776	1624 (0 to 6278)	0.001	0.003^†^
SKVF, III4e area (°^2^)	0 (−208 to 3917)	0.321	3286 (1274 to 5641)	<0.001	0.074
SKVF, V4e area (°^2^)	471 (−36 to 3150)	0.202	1886 (332 to 4360)	0.016	0.584
FST, white (log cd·s/m^2^)^‡^	−2.79 (−3.75 to −1.96)	<0.001	−2.86 (−3.5 to −2.27)	<0.001	0.628
FST, blue (log cd·s/m^2^)^‡^	−3.3 (−4.25 to −2.22)	<0.001	−2.88 (−4.01 to −2.28)	<0.001	0.969
FST, red (log cd·s/m^2^)^‡^	−1.49 (−2.35 to −1.07)	0.023	−1.22 (−1.46 to −0.67)	<0.001	0.334

* Compared to baseline.

† Comparison between groups showed a statistically significant difference with significant improvement compared to baseline only in the group of eyes with touchdown or no CRA.

‡ More negative FST threshold values indicate improved retinal sensitivity.

## Discussion

This retrospective longitudinal study of 41 consecutive eyes from 25 patients with *RPE65*-mediated IRDs treated with VN therapy is one of the largest European case series reported by a single center to date. Our results showed a significant improvement in BCVA over the follow-up (median change = −0.20 logMAR, corresponding to 10 ETDRS letters), which is in line with the phase III clinical trial outcomes (i.e., 11 and 8 ETDRS letters in the first and second treated eye, respectively) and the real-life reports (ranging from 5 to 12 ETDRS letters).^6^^,^^7^^,^^9^^,^^10^^,^^13^^,^^18^^,^^19^

Moreover, a significant visual field enlargement was noted, particularly when evaluated with the I4e and III4e stimulus sizes. This observation is consistent with data from the phase III treated subjects^5^ and from the previous real-life study by Deng et al.^22^ However, it should be noted that these studies measured the sum of the radii along the 24 meridians and adopted manual Goldmann perimeters, which are more user dependent compared to the automated perimeter used in this study.

FSTs significantly improved in response to white (−2.79 log cd·s/m^2^) and blue (−3.02 log cd·s/m^2^) stimuli, in line with the 2.3 log cd·s/m^2^ decrease observed in the phase I follow-on and phase III trial reports^4^^,^^5^ and rather higher than the improvement range of −1.32 to −2.41 log cd·s/m^2^ described in different real-life studies.^13^^,^^17^^,^^22^ Furthermore, we also observed a slight improvement (−1.3 log cd·s/m^2^) in the FSTs to red stimuli, even if the change was smaller. Overall, these findings confirmed the beneficial effects of VN on both rod- and cone-mediated visual function, as previously suggested by Lorenz et al.^17^

Furthermore, retinal imaging revealed the development of different CRA patterns in 15 patients (60%), including a focal area of atrophy corresponding to the retinotomy site in six patients, a nummular pattern in eight patients, and a mixed pattern in one patient, first evident at the 6-month time point. It is important to acknowledge that CRA is a well-documented component of the natural progression of *RPE65*-related IRDs, particularly in older patients who have been followed longitudinally prior to the availability of VN. In these studies,^7^^–^^10^ atrophic changes such as peripheral lacunar lesions, peripapillary hypopigmentation, and macular RPE mottling have been reported to emerge in the second decade of life and progress slowly over years to decades. For this reason, natural progression can make it challenging to distinguish between atrophy related to ongoing disease and that related to treatment. However, several features observed in our cohort of treated patients suggest that the CRA detected after VN treatment appears to differ from that typically reported in natural disease progression. First, CRA in our cohort of treated eyes predominantly occurred in children and young adults, whereas atrophic changes described in natural history studies^7^^–^^10^ have more commonly been reported in older patients. Second, the spatial distribution of atrophy in our cohort showed a consistent topographic relationship with surgical landmarks, corresponding either to the retinotomy site (touchdown pattern) or, at least partially, to the area exposed to the subretinal bleb (nummular/mixed patterns), as shown in Figures 1 and 2. Notably, these lesions were often located along the vascular arcades, whereas natural history reports described atrophic changes typically observed in more peripheral retinal regions.^7^^–^^10^ In addition, the temporal profile of CRA development in our series was characterized by an early onset—already evident within 6 months after treatment—and by a measurable enlargement over the subsequent follow-up interval. This relatively rapid evolution contrasts with the slower and more gradual progression of atrophy generally reported in untreated *RPE65*-related disease. In this regard, in our multicentric natural history study,^7^ including 35 patients followed up for a median period of 4.6 years, we did not observe any nummular or mixed CRA, and it is notable that most of our treated patients (13 out of 25, 52%; ID 2, 3, 4, 5, 6, 7, 8, 9, 10, 11, 12, 19, 20) belonged to this cohort. Taken together, although we cannot exclude that some components of CRA may overlap with the natural course of the disease, these differences in patient age, topographic distribution, and temporal dynamics suggest that the observed atrophic changes are unlikely to be solely attributable to natural disease progression and may instead be influenced by treatment-related factors, including surgical manipulation or subretinal vector delivery.

In agreement with Bommakanti et al.,^15^ we observed over our 1-year follow-up a significant growth of the nummular and mixed CRA areas and a reduced progression of touchdown CRA after VN treatment. Bommakanti et al.^15^ suggested that touchdown CRA may be triggered by different causes compared to the other patterns of atrophy. On the basis of this consideration, we analyzed risk factors and treatment effects in our cohort comparing patients with a progressive pattern of CRA (i.e., nummular or mixed) and those with touchdown atrophy or no CRA occurrence. Our results showed that significant improvements in BCVA and FSTs occurred in both of the patient groups. In contrast, the enlargement of visual field, particularly with the I4e stimulus size, was significantly lower in patients with nummular/mixed CRA patterns compared to those with touchdown CRA or no atrophy. Although various studies have investigated kinetic or static perimetry after VN treatment,^17^^,^^22^^,^^23^ the comparison of these findings is limited because, to our knowledge, only a few previous studies^6^^,^^11^ have investigated the associations between visual field changes and the development of CRA. In particular, one study did not identify significant correlations but reported only visual fields evaluated with the V4e stimulus size, which may not be sensitive enough in the detection of treatment effects.^11^ Additionally, Gange et al.^6^ reported changes in the visual fields of eyes that developed CRA, but they did not provide a comparison with eyes without CRA.

Moreover, patients showing progressive nummular and mixed CRA were on average younger and had less advanced retinal degeneration when compared to those without CRA, for example showing more frequently a detectable EZ band. The association of CRA with younger age and with non-advanced retinal degeneration has been recently explored by Stingl et al.,^12^ who postulated that CRA may develop as a result of a sudden metabolic turnover of the degenerated retina due to restoration of the photoreceptor function, especially in pediatric patients. They demonstrated that retinal areas with the greatest gain of rescued rods after treatment are more prone to be the initial spots of atrophy. However, these results are based on two retinotopic tests that are not routinely used in clinical practice (i.e., dark-adapted chromatic perimetry and scotopic chromatic pupil campimetry), thus limiting comparison with other studies. The bilateral occurrence of CRA and its presence in two pairs of siblings in our cohort suggests that both patient-specific and procedure-related factors (e.g., genotype, individual retinal vulnerability, immune response to the vector, surgical stress) may also contribute to CRA development following VN treatment. Surgical factors may impact outcomes, as well. In our cohort, eyes experiencing intraoperative foveal detachment showed a higher frequency of progressive nummular/mixed CRA compared to eyes without foveal detachment. Although this association did not reach statistical significance after accounting for inter-eye correlation in the GEE model, the observed trend suggests that intraoperative foveal detachment may represent a clinically relevant risk factor. Therefore, this finding may pose potential surgical implications and warrants further investigation in larger cohorts of patients.

The strengths of the current study include the single-center and single-surgeon setting without significant intraoperative complications, as well as a standardized imaging and treatment protocol for all treated patients. On the other hand, this study has several limitations. The 12-month follow-up may not have captured the long-term evolution of CRA or its functional impact, and the relatively small sample size—reflecting the rarity of *RPE65*-related IRDs—limits the power of group comparisons. The retrospective design also introduces variability in data completeness. Assessment of EZ integrity was less reliable in patients with significant nystagmus, in whom only single foveal B-scans could be obtained, potentially leading to misclassification. Although CRA lesions were independently segmented by two expert graders with full agreement on lesion count and boundaries, formal interobserver agreement metrics were not calculated, representing an additional methodological limitation. Finally, CRA extent could not be spatially correlated with visual field defects because SKVF lacks the retinotopic alignment with fundus imaging.

## Conclusions

Our findings confirm the improvements in visual function (i.e., BCVA, SKVF, and FSTs), consistent with both clinical trial data^4^^,^^5^ and recent studies in clinical practice,^11^^–^^15^ and they support safety and effectiveness of VN treatment in patients with *RPE65*-related IRDs. Importantly, despite the relatively high (60%) prevalence of CRA observed after gene therapy, improvements in BCVA and FSTs were preserved, indicating that CRA development does not prevent the overall functional benefit of the treatment. Moreover, for the first time, we showed that SKVF using a smaller stimulus size (i.e., I4e) may effectively estimate the negative impact of nummular and mixed CRA on improvement of visual function. Furthermore, the association between CRA development, younger age, and a detectable EZ band before treatment suggests that metabolic overstimulation following VN gene therapy may contribute to the development of progressive CRA patterns. Nonetheless, other factors, including surgical-related stress or inflammatory mechanisms, cannot be excluded, particularly in cases of touchdown atrophy. These findings underscore the need for careful monitoring of CRA patterns as potential long-term sequelae of VN therapy and highlight the importance of integrating sensitive functional endpoints in post-treatment follow-up. Ultimately, this information may support more informed patient selection and counseling regarding the expected benefits and risks. Larger prospective studies with extended follow-up are warranted to further elucidate CRA risk factors and their long-term functional implications.
